# Conditioning regimens for allogeneic hematopoietic cell transplantation in acute myeloid leukemia: Real-world data from the Japanese registry studies

**DOI:** 10.3389/fonc.2022.1050633

**Published:** 2022-11-25

**Authors:** Masamitsu Yanada, Kaito Harada, Yoshimitsu Shimomura, Yasuyuki Arai, Takaaki Konuma

**Affiliations:** ^1^ Department of Hematology and Cell Therapy, Aichi Cancer Center, Nagoya, Japan; ^2^ Department of Hematology and Oncology, Tokai University School of Medicine, Isehara, Japan; ^3^ Department of Hematology, Kobe City Medical Center General Hospital, Kobe, Japan; ^4^ Department of Hematology and Oncology, Graduate School of Medicine, Kyoto University, Kyoto, Japan; ^5^ Department of Hematology/Oncology, The Institute of Medical Science, The University of Tokyo, Tokyo, Japan

**Keywords:** acute myeloid leukemia, allogeneic hematopoietic cell transplantation, conditioning regimen, myeloablative conditioning, reduced-intensity conditioning

## Abstract

Conditioning regimens play a crucial role in preventing relapse of acute myeloid leukemia (AML) following allogeneic hematopoietic cell transplantation (HCT). In early times, myeloablative conditioning was used exclusively, but it was associated with significant toxicity. However, the advent of reduced-intensity conditioning has allowed allogeneic HCT to be performed more safely, leading to an expansion of our choices for conditioning regimens. As the transplantation methods have become highly diversified, it is reasonable to determine an optimal conditioning regimen in consideration of patient-, disease-, and transplantation-related factors. In this context, large-scale registry-based studies provide real-world data to allow for a detailed evaluation of the utility of individual conditioning regimens in specific clinical settings. The Japanese Society for Transplantation and Cellular Therapy has been conducting a nationwide survey for HCT since 1993 that currently covers >99% of all the transplantation centers nationwide, and >1,000 allogeneic HCTs performed for adults with AML are registered per year. We have been using the registry data to implement a number of studies focusing on adults with AML, and the large number of patients registered consecutively from nearly all transplantation centers nationwide represent real-world practice in Japan. This article reviews and discusses the results obtained from our registry-based studies pertaining to various conditioning regimens.

## Introduction

Allogeneic hematopoietic cell transplantation (HCT) is the most potent therapy for preventing relapse of acute myeloid leukemia (AML), in which conditioning regimens play a pivotal role in eradicating leukemic cells (1–4). Previously, myeloablative conditioning (MAC) was commonly administered, but the high toxicity limited its use. However, the advent of reduced-intensity conditioning (RIC) in the late 1990s has allowed allogeneic HCT to be performed more safely, thus expanding the applicability of this procedure as well as our choices for conditioning regimens (5, 6). Although several prospective randomized studies have been conducted that compared different conditioning regimens (7–10), uncertainty persists regarding optimal regimens for individual patients. To address this issue, large-scale registry-based studies are expected to provide the requisite real-world data to complement results obtained from prospective randomized studies.

In 1993, the Japanese Society for Transplantation and Cellular Therapy (JSTCT) launched a nationwide survey regarding HCT wherein HCTs performed during the previous year in participating centers have been consecutively registered, and this registration program currently covers >99% of all the transplantation centers nationwide. AML represents the most common indication for allogeneic HCT, accounting for approximately 50% in adults (11), with >1,000 allogeneic HCTs performed for adults with AML being registered per year (12). The Adult AML Working Group of the JSTCT has been using the registry data to investigate various aspects of HCT for AML (13). Herein, we review and discuss the results obtained from our registry-based studies pertaining to various conditioning regimens.

## Distribution of conditioning regimens in Japan

**Figure 1:**
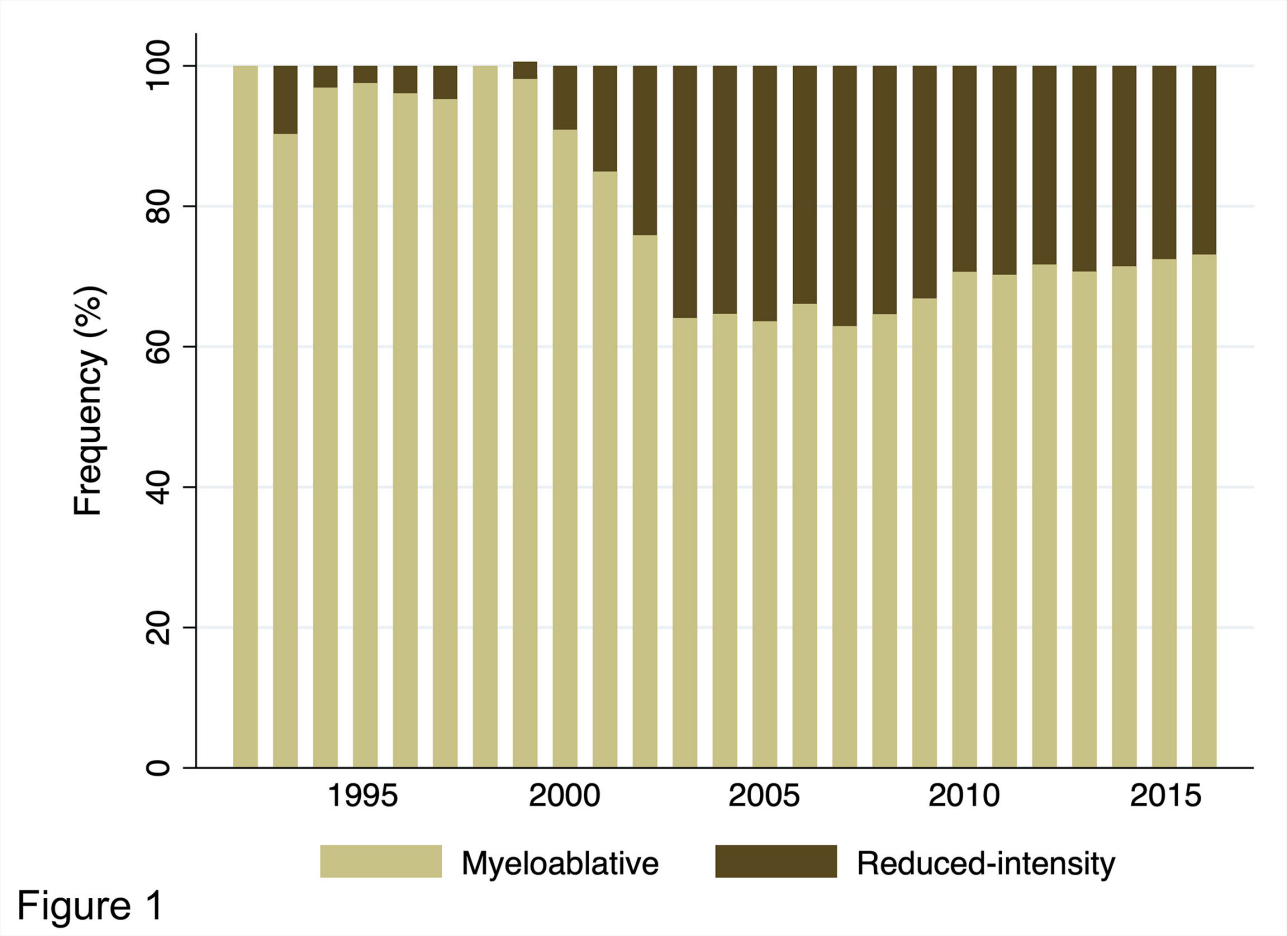
Annual trends in the distribution of conditioning intensity for adults with AML undergoing allogeneic HCT from 1992 to 2016 in Japan.

**Figure 1** shows the annual changes in the rates of MAC and RIC use for adults (age ≥16 years, the general threshold above which patients are treated by hematologists in Japan) with AML who underwent their first allogeneic HCT from 1992 to 2016. For our registry data, conditioning regimens were defined as MAC if either total body irradiation (TBI) >8 Gy, oral busulfan ≥9 mg/kg, intravenous busulfan ≥7.2 mg/kg, or melphalan >140 mg/m^2^ was used; otherwise, they were considered RIC (14). The use of RIC began to increase from 2000 and peaked around 2005; its usage rate remains at approximately 30% in recent years. Details of the conditioning regimens are summarized in **Table 1**. Overall, MAC and RIC were used for 9,976 (71%) and 4,034 (29%) patients, respectively. Of the patients conditioned with MAC, the cyclophosphamide plus total body irradiation (CY/TBI)-based regimen accounted for 51% of all regimens, whereas the busulfan plus cyclophosphamide (BU/CY)-based regimen accounted for 18%. As for RIC, the fludarabine plus busulfan (FLU/BU)-based, the fludarabine plus melphalan (FLU/MEL)-based, and the fludarabine plus cyclophosphamide (FLU/CY)-based regimens were administered to 41%, 40%, and 8% of patients, respectively. These statistics highlight unique features of RIC regimens used in Japan, characterized by the popularity of melphalan comparable to that of busulfan and the low prevalence of so-called nonmyeloablative conditioning regimens.

**Table 1:** Distribution of conditioning regimens for adults with AML undergoing allogeneic HCT during the period 1992 to 2016 in Japan.

Myeloablative conditioning	No. of patients	%
CY/TBI-based	5144	51%
Other TBI-based	604	6%
BU/CY-based	1767	18%
Other non-TBI-based	2461	25%
***Reduced-intensity conditioning***	***No. of patients***	***%***
FLU/BU-based	1660^a^	41%
FLU/MEL-based	1619	40%
FLU/CY-based	333	8%
Others	422	11%

CY, cyclophosphamide; TBI, total body irradiation; BU, busulfan; FLU, fludarabine; MEL, melphalan.

a Including 178 patients conditioned with FLU/BU/MEL.

## Myeloablative conditioning

CY/TBI and BU/CY represent the two most common MAC regimens. Several randomized studies were conducted to compare these two regimens for patients with various diseases, including AML, but the results are conflicting (15–18). A meta-analysis of these prospective randomized studies did not find any significant difference in disease-free and overall survival (OS) between the two regimens (19). The subsequent development of an intravenous formulation of busulfan (ivBU) mitigated inter-patient differences in absorption and metabolism inherent to the oral formulation of busulfan (poBU), allowing for better control of plasma busulfan levels (20). Retrospective studies conducted by the European Society for Blood and Marrow Transplantation (EBMT) and the Center for International Blood and Marrow Transplantation Research (CIBMTR) showed that ivBU/CY is preferred over poBU/CY and is either comparable to or even better than CY/TBI (21, 22). By using the nationwide registry data, we compared CY/TBI, poBU/CY, and ivBU/CY for 3,667 adults with AML (23). Multivariate analysis revealed that ivBU/CY was associated with a lower risk of non-relapse mortality (NRM) than CY/TBI (hazard ratio [HR], 0.68; 95% confidence interval [CI], 0.51–0.90; P = 0.007) or poBU/CY (HR, 0.60; 95% CI, 0.43–0.83; P = 0.002), corroborating the less toxic profile of ivBU/CY. In contrast, there was no difference in the relapse rate or OS between the conditioning regimens.

With the aim of enhancing antileukemic effect, high-dose cytarabine may be incorporated into CY/TBI especially in patients with features of high-risk disease features. We conducted two studies to evaluate the effectiveness of adding high-dose cytarabine into CY/TBI (CA/CY/TBI). The first study analyzed 929 patients undergoing umbilical cord blood transplantation (UCBT) with CY/TBI or CA/CY/TBI, which demonstrated that the addition of high-dose cytarabine contributed to lower overall mortality (HR, 0.56; 95% CI, 0.45–0.69; P < 0.01) through a reduction in relapse (HR, 0.50; 95% CI, 0.38–0.67; P < 0.01) without increasing NRM (HR, 0.94; 95% CI, 0.67–1.33; P = 0.73) (24). Moreover, a higher dose of cytarabine (12 g/m^2^ vs. 8 g/m^2^ in total) was found to correlate with better OS for patients with high-risk disease but not for those with standard-risk disease. The second study analyzed 2,102 patients undergoing allogeneic bone marrow transplantation (BMT) or peripheral blood stem cell transplantation (PBSCT) (25). In contrast to the first study, the addition of high-dose cytarabine was not associated with improved outcomes; leukemia-related mortality did not decrease (HR, 0.90; 95% CI, 0.72–1.12; P = 0.34), and NRM turned out to be significantly higher (HR, 1.48; 95% CI, 1.15–1.91; P < 0.01), resulting in a non-significant trend toward a worse OS for patients receiving CA/CY/TBI than for those receiving CY/TBI alone (HR, 1.14; 95% CI, 0.96–1.34; P = 0.13).

Although fractionated TBI at a total dose of 12 Gy is standard when incorporated into a MAC regimen (26), there is a paucity of data on whether TBI fractionation has a significant effect on posttransplant outcomes. Thus, we performed a study to evaluate the optimal number of fractions for patients with AML undergoing allogeneic HCT following a conditioning regimen that included 12 Gy of TBI (27). Among the 4,050 patients, TBI was delivered almost exclusively in four (n = 1,215, 30%) or six fractions (n = 2,697, 67%), leading to comparisons between 4- versus 6-fraction TBI. Compared to 6-fraction TBI, 4-fraction TBI was associated with a reduced risk of overall mortality (HR, 0.85; 95% CI, 0.77–0.95; P = 0.003) and relapse (HR, 0.86; 95% CI, 0.75–0.98; P = 0.021). The subgroup analysis revealed that 4-fraction TBI had a significantly positive prognostic impact for patients in non-complete remission (CR) at the time of transplantation, suggesting the advantage of 4-fraction over 6-fraction TBI for patients at high risk of posttransplant relapse. In contrast, an analysis of the EBMT registry data found that patients who had received 12-Gy TBI in three to four fractions showed a similar risk of relapse to those who had received 12-Gy TBI in six fractions (28). This discrepancy could be explained by a small proportion of patients in non-CR included in the EBMT study (14% of the total), considering that only patients in non-CR in our study showed a significant difference in outcomes by 4- versus 6-fraction (27).

## Reduced-intensity conditioning

In our registry, the percentage of patients aged ≥60 years has been increasing since 2000, which was when RIC was coincidentally introduced (12). To investigate whether outcomes of RIC allogeneic HCT have changed over time, we analyzed data of 2,325 patients aged >50 years with AML who underwent RIC allogeneic HCT between 2000 and 2013 (29). When the year of transplantation was divided into two periods (2000–2007 and 2008–2013), patients who underwent transplantation during the latter period had better OS (39% vs. 32% at 3 years, P < 0.001) and lower NRM (38% vs. 46% at 3 years, P < 0.001) than those who underwent transplantation during the earlier period. Another study regarding older patients who underwent RIC allogeneic HCT showed that neither OS nor NRM differed for any of the age groups, 50–54, 55–59, 60–64, and ≥65 years, which is in accordance with an EBMT study (30), suggesting that advanced age alone should no longer be considered a contraindication for allogeneic HCT (31).

Unlike older patients, data are scarce for the clinical utility of RIC allogeneic HCT in younger patients as MAC remains the conditioning of choice for these populations. Therefore, we analyzed outcomes of 125 patients with AML <50 years old who underwent RIC allogeneic HCT (32). In the univariate analysis, patients who received RIC had significantly worse OS than those who received MAC (48% vs. 54% at 4 years, P = 0.047). However, this difference was only marginally significant after adjusting for baseline patient characteristics; the propensity score-based matched pair analysis further diminished the significance, indicating the existence of a selection bias against RIC. Despite such a selection bias, the observed 4-year OS of 48% with RIC allogeneic HCT was still acceptable, rendering support for the use of RIC in younger patients who are considered unsuitable for MAC. Notably, subgroup analysis in this study showed similar outcomes between RIC and MAC for patients aged between 40 and 49 years and those in first or second CR at the time of transplantation, which sharply contrasts with the finding that their counterparts, i.e., those <40 years old and those who were in advanced disease at the time of transplantation, showed significantly inferior OS with RIC.

Some of the studies compared different RIC regimens. As previously mentioned, FLU/MEL and FLU/BU are the two most common RIC regimens in Japan, and their frequencies of clinical use are almost equal. After discriminating between the intravenous and oral formulations of busulfan, we conducted a three-group comparison of FLU/MEL, FLU/ivBU, and FLU/poBU (33). In the multivariate analysis using FLU/ivBU as the reference category, FLU/MEL was associated with a lower risk of relapse (HR, 0.65; 95% CI, 0.50–0.85; P = 0.002) and a higher risk of NRM (HR, 1.60; 95% CI, 1.10–2.33; P = 0.013), whereas FLU/poBU was associated with a higher risk of NRM (HR, 1.84; 95% CI, 1.28–2.64; P = 0.001). In terms of OS, no significant difference was found between the groups. These results suggest that both FLU/MEL and FLU/ivBU are useful RIC regimens, with FLU/MEL being characterized by more potent antileukemic activity and FLU/ivBU by lower toxicity. The EBMT registry data showed similar findings; FLU/MEL was associated with a lower relapse rate and a trend for higher NRM than FLU/BU, resulting in similar OS (34).

FLU/MEL was originally developed with a total dose of 140 mg/m^2^ for melphalan (35), but a lower dose is sometimes used in practice because of concerns about toxicity. Our study focusing on the melphalan dose in FLU/MEL showed that patients who received a higher dose (120–140 mg/m^2^) had lower leukemia-related mortality (HR, 0.50; 95% CI, 0.30–0.85; P = 0.01), similar NRM (HR, 0.87; 95% CI, 0.56–1.35; P = 0.53), and lower overall mortality (HR, 0.63; 95% CI, 0.46–0.88; P < 0.01) than those who received a lower dose (80–110 mg/m^2^) (36). The survival advantage with the use of a higher dose was evident in patients <60 years old, those who underwent transplantation in non-CR, and those with good performance status.

Low-dose TBI is frequently added to a RIC regimen to facilitate donor cell engraftment through inhibition of the host immune response. We thus examined whether the addition of low-dose TBI (≤4 Gy) was beneficial in 409 patients undergoing RIC allogeneic HCT from HLA-matched related donors (37). The addition of low-dose TBI did not improve neutrophil or platelet engraftment, and no difference was found in terms of OS, relapse, or NRM for those conditioned with and without low-dose TBI, indicating no significant benefits with the addition of low-dose TBI in HLA-matched related transplantation. Meanwhile, the effects of low-dose TBI for allogeneic HCT from alternative donors are being investigated in an ongoing study.

## Comparisons of myeloablative versus reduced-intensity conditioning

Retrospective studies comparing the efficacy of RIC and MAC in AML showed inconsistent results (38–43), with some reporting a higher relapse rate (38–40) and lower NRM (38, 40–42) for patients conditioned with RIC. Several prospective randomized studies have compared conditioning regimens of different intensities. Bornhauser et al. compared CY/TBI at a total radiation dose of 8 vs. 12 Gy for patients with AML, and reported no significant differences in relapse, NRM, or OS (7). Scott et al. randomized patients with AML or myelodysplastic syndrome (MDS) to RIC (mostly FLU/BU) or MAC and observed better relapse-free survival with MAC (8). Kröger et al. compared RIC (FLU/BU) with MAC for patients with MDS or secondary AML and found no difference in outcomes (9). Craddock et al. showed that their augmented RIC regimen (defined as MAC based on our definitions) resulted in similar relapse, NRM, and OS compared with the standard RIC regimens (10). These conflicting data preclude a definitive conclusion on the relative merits of RIC and MAC. Furthermore, most of these studies are limited by a relatively short follow-up duration. Therefore, we attempted to elucidate the long-term outcomes of patients aged ≥50 years with AML who underwent RIC (n = 284) or MAC (n = 190) allogeneic HCT during CR (44). Based on >10 years of follow-up data, RIC and MAC recipients had similar outcomes in terms of OS (36% vs. 40% at 10 years, P = 0.752), relapse (30% vs. 26% at 10 years, P = 0.420), and NRM (36% vs. 36% at 10 years, P = 0.906). This study confirmed the long-term efficacy of RIC allogeneic HCT as it had an equivalent OS to that of MAC without an increase in the rate of late relapse. Shimoni et al. on behalf of the EBMT also reported equivalent long-term OS for patients receiving RIC and MAC by analyzing their data securing a median follow-up duration of 8.3 years (42).

The choice between RIC and MAC is largely determined by patient-related factors such as age, performance status, and comorbidities; however, the optimal conditioning intensity may also depend on disease-related factors. A subgroup analysis of a randomized study by Scott et al. suggests a survival advantage for MAC over RIC in patients with high-risk disease as defined by unfavorable cytogenetics, presence of *FLT3* mutation, or third or subsequent CR (8). Cytogenetic risk is one of the most important disease-related factors in AML that predicts outcomes following allogeneic HCT (45, 46). By focusing on AML with poor-risk cytogenetics, we evaluated the effect of conditioning intensity for 840 such patients undergoing allogeneic HCT during first CR (47). In this cohort of patients with an adverse prognosis, MAC was found to be superior to RIC in terms of OS (54% vs. 40% at 3 years, P < 0.001) and leukemia-related mortality (21% vs. 31% at 3 years, P = 0.007). Investigators from the EBMT and those from the CIBMTR also analyzed outcomes of allogeneic HCT during first CR for patients with AML harboring unfavorable cytogenetics (48, 49). The EBMT study showed no significant effect of conditioning intensity on OS (48), whereas the CIBMTR study reported superior OS in patients older than 50 years (49). Besides cytogenetics, recent studies have shown the prognostic significance of measurable residual disease (MRD) prior to transplantation (50, 51). We assessed outcomes of patients with t (8, 21) and inv (16) AML who underwent allogeneic HCT during CR in relation to pretransplant MRD status as measured by a polymerase chain reaction assay of the *RUNX1-RUNX1T1* or *CBFB-MYH11* fusion transcripts (52). An analysis of 959 patients showed that the conditioning intensity did not affect relapse or OS in patients with t (8, 21) irrespective of the MRD status, whereas MAC provided better OS for those with inv (16) AML only if their pretransplant MRD was negative.

## Conditioning regimens for umbilical cord blood transplantation

Umbilical cord blood (UCB) is an alternative donor source for patients requiring allogeneic HCT who lack a matched related and unrelated donor. UCBT has been performed actively in Japan; >1,000 single-unit UCBTs are performed per year all over the country, which accounts for one-third of all allogeneic HCTs (12). The primary reason for the widespread use of UCBT in Japan is the feasibility of finding a suitable UCB unit, which can be partly explained by the relatively small body size of Japanese patients, thus permitting a lower cell dose, as well as less stringent criteria for UCB unit selection than those used outside Japan (53).

Given that most UCBT patients receive an HLA-mismatched UCB unit containing a lower cell dose compared to those undergo allogeneic BMT or PBSCT, engraftment failure is a significant concern. Hence, more intensive conditioning regimens may be beneficial to overcome this shortcoming. In Japan, a temporal increase in the use of MAC regimens was observed, as the proportions of MAC regimens were 57% in 1998–2007, 62% in 2008–2013, and 71% in 2014–2019 for patients with AML undergoing UCBT (54). The data of 5,504 patients with AML undergoing UCBT between 1998 and 2019 showed that RIC was associated with a higher risk of relapse-related mortality compared to MAC (HR, 1.14; 95% CI, 1.02–1.28; P = 0.021), but there was no difference between RIC and MAC in terms of overall mortality (HR, 1.03; 95% CI, 0.95–1.13; P = 0.386) or NRM (HR, 0.93; 95% CI, 0.82–1.06; P = 0.300) (54).

Due to its immunosuppressive effects that prevent graft rejection, TBI-based conditioning regimens are widely used for UCBT, among which CY/TBI is a representative MAC regimen. Against such a background, we evaluated whether combining high-dose cytarabine with CY/TBI (CA/CY/TBI) improves UCBT outcomes. As mentioned earlier, the addition of high-dose cytarabine to CY/TBI was associated with lower relapse and similar NRM, resulting in better OS (24).

The administration of granulocyte-colony stimulating factor (G-CSF) increases the susceptibility of leukemic cells to cytarabine through the induction of cell cycle entry of dormant leukemia cells (55). This rationale prompted us to investigate whether concurrent administration of G-CSF with a conditioning regimen improves UCBT outcomes, and the study results showed that G-CSF combined with CA/CY/TBI resulted in faster neutrophil engraftment, lower relapse, and better OS (56). Following these encouraging results, we are now conducting a prospective randomized study to compare CA/CY/TBI with or without concurrent administration of G-CSF for patients with AML or MDS undergoing UCBT (57).

Despite positive features of TBI as conditioning for UCBT, it causes significant organ toxicities and secondary malignancies (26). Furthermore, some centers may have difficulties in delivering TBI in a timely manner because of logistical reasons. Yamamoto et al. recently developed a novel non-TBI regimen for UCBT consisting of fludarabine, busulfan, and melphalan (FLU/BU4/MEL), and they reported durable engraftment, acceptable toxicity, and significant antileukemic effects in patients with advanced myeloid malignancies (58). The promising results of this single-center study have rapidly expanded the use of this regimen across the country. In addition, the utility of FLU/BU4/MEL in UCBT has been validated in a large number of patients enrolled in the nationwide registry, which is described in detail in the next section.

## Novel conditioning regimens

A novel conditioning regimen, FLU/BU4/MEL, was developed to overcome the limitations associated with FLU/BU4. Although FLU/BU4 is currently one of the standard MAC regimens, relatively high rates of posttransplant relapse pose a significant challenge especially for patients with advanced disease (59, 60). Meanwhile, the low toxicity profile of FLU/BU4 allows for an additional chemotherapeutic agent to be combined, which may contribute to a reduction in relapse. Melphalan is considered a suitable option to be combined with FLU/BU4 by virtue of the possible synergistic effect and the different toxicity profile (61, 62). A single-center prospective study evaluated the efficacy and safety of FLU/BU4/MEL for 51 patients with advanced myeloid malignancies who underwent UCBT. For this study, melphalan was administered at 40 mg/m^2^ for 2 days in combination with fludarabine at 30 mg/m^2^ for 6 days and intravenous busulfan at 3.2 mg/kg for 4 days, which forms the basis for the currently widely-used FLU/BU4/MEL regimen. The results were encouraging, with 2-year probabilities of OS, NRM, and relapse of 55%, 26%, and 20%, respectively (58). Another single-center study retrospectively analyzed the efficacy and safety of FLU/BU4/MEL for 42 patients with AML and MDS who underwent allogeneic BMT or PBSCT, and showed that the 4-year OS, NRM, and relapse were 66%, 19%, and 21%, respectively (63).

Subsequently, data from the Japanese nationwide registry were analyzed to compare FLU/BU4/MEL with FLU/BU4 (64). This study included 846 propensity score-matched patients who received either FLU/BU4/MEL or FLU/BU4, the majority of whom had AML (71%) and high-risk disease (61%). The 5-year OS was 34% in the FLU/BU4/MEL group versus 30% in the FLU/BU4 group (P = 0.019). The better OS in the FLU/BU4/MEL group was attributable to the lower relapse rate, and there was no difference in NRM between the groups. More recently, FLU/BU4/MEL was compared with conventional MAC regimens including BU/CY and CY/TBI for patients with relapsed or refractory AML (65). This study also used a propensity score-matching method to identify 188 patients (94 pairs) with relapsed or refractory AML whose demographics were well balanced. The 5-year OS was 45% in the FLU/BU4/MEL group versus 24% in the conventional MAC group (P = 0.002). FLU/BU4/MEL was found to be associated with a lower risk of overall mortality (HR, 0.57; 95% CI, 0.36–0.90; P = 0.015), a lower risk of relapse (HR, 0.64; 95% CI, 0.42–0.96; P = 0.031) and had a similar risk of NRM (HR, 0.68; 95% CI, 0.35–1.31; P = 0.250) compared to the conventional MAC regimens.

The efficacy of FLU/BU4/MEL was evaluated in 477 patients with AML undergoing UCBT during CR; 106, 148, and 223 patients received FLU/BU4/MEL, CY/TBI, and CA/CY/TBI, respectively (66). Patients in the FLU/BU4/MEL group were older, had higher HCT-specific comorbidity index scores, and were more likely to harbor poor cytogenetics compared to the other groups. In the univariable analysis, there was no difference in the 3-year OS (66% vs. 65% vs. 65% at 3 years, P = 0.71), relapse (18% vs. 22% vs. 17% at 3 years, P = 0.40), or NRM (19% vs. 17% vs. 21% at 3 years, P = 0.95). However, the multivariate analysis revealed superior OS with FLU/BU4/MEL compared to CY/TBI (HR, 0.50; 95% CI, 0.29–0.88; P = 0.015) and CA/CY/TBI (HR, 0.57; 95% CI, 0.32–1.01; P = 0.052).

The results described above support the usefulness of FLU/BU4/MEL in AML, and needs to be confirmed in a prospective randomized study. Note that several agents used outside Japan such as clofarabine, amsacrine, treosulfan, and radio-immuno conjugates are not approved for use in conditioning in Japan and thus are not used in clinical practice.

## Discussion

Although AML represents the most common indication for allogeneic HCT, limited numbers of patients as well as the highly complex nature of the procedure often make it difficult to conduct a prospective randomized study to compare different conditioning regimens. In addition, it is important to note that patients entered into a prospective randomized study are fit enough to meet predefined inclusion and exclusion criteria; thus, they do not represent the general patient population. From this point of view, registry studies provide real-world information and allow for a detailed evaluation of the utility of individual conditioning regimens in specific clinical settings. On the other hand, we should keep in mind potential limitations associated with the retrospective nature of registry studies, including selection bias, patient and treatment heterogeneity, and the lack of detailed data on certain variables. Statistical methods, such as multivariate analysis and propensity score-matching analysis, facilitate adjustments for known confounding factors, but there may well be possible unknown or unmeasured factors that may influence study results. As an example, our studies published so far have not considered specific mutations such as those of *FLT3*, *NPM1*, and *TP53* because our registry systematically started collecting such information in 2019.

Acknowledging these limitations, focusing on AML and the large number of patients constitute the unique strengths of our registry-based studies. **Table 2** provides a brief summary of our studies described earlier. As the transplantation methods become highly diversified, the concept of “one-conditioning-fits-all” becomes less applicable, and it is reasonable that the optimal conditioning regimen needs to be determined with consideration given to patient-, disease-, and transplantation-related factors including age, performance status, comorbidities, disease and disease status, and donor source. Therefore, it would be difficult to make generalized recommendations on the choice of conditioning regimens based on the findings of our studies. However, well-designed large-scale retrospective analyses may have the potential to provide the best available evidence to aid clinical decision making, and it is hoped that the registry-based studies referenced above help in the optimization of conditioning regimens for allogeneic HCT in adults with AML and that currently unsettled issues will be addressed by ongoing and future studies.

**Table 2:** Summary of the selected Japanese registry studies.

Study	N	Objective	Key finding
Yamashita et al., 2013 (23)	MAC, 3667	Comparing CY/TBI and BU/CY	ivBU/CY was associated with lower NRM than CY/TBI and poBU/CY.
Arai et al., 2015 (24)	MAC, 929	Evaluating the effectiveness of adding high-dose cytarabine to CY/TBI in UCBT	The addition of high-dose cytarabine reduced relapse and improved OS without increasing NRM.
Arai et al., 2015 (25)	MAC, 2102	Evaluating the effectiveness of adding high-dose cytarabine to CY/TBI in BMT or PBSCT	The addition of high-dose cytarabine did not reduce relapse or improve OS, but increased NRM.
Ueda et al., 2021 (27)	MAC, 3912	Comparing 4- versus 6-fraction for 12-Gy TBI administration	Patients transplanted in non-CR benefited from 4-fraction over 6-fraction.
Yamasaki et al., 2017 (29)	RIC, 2325	Evaluating the survival trends for patients older than 50 years undergoing RIC allogeneic HCT	OS and NRM improved over time during the period 2000–2013.
Aoki et al., 2016 (31)	RIC, 757	Evaluating the effect of age on outcomes following RIC allogeneic HCT for patients older than 50 years	Neither OS nor NRM differed for patients aged 50–54, 55–59, 60–64, and ≥ 65 years.
Yanada et al., 2017 (32)	MAC, 1554 RIC, 125	Evaluating outcomes following RIC allogeneic HCT for patients younger than 50 years	The survival advantage for MAC over RIC disappeared after adjustment for patient characteristics.
Yamashita et al., 2020 (33)	RIC, 1221	Comparing FLU/BU and FLU/MEL in RIC allogeneic HCT	Compared to FLU/ivBU, FLU/MEL was associated with a lower risk of relapse and a higher risk of NRM, whereas FLU/poBU was associated with a higher risk of NRM.
Harada et al., 2019 (36)	RIC, 507	Evaluating the prognostic impact of melphalan dose in RIC allogeneic HCT	A total melphalan dose of 120–140 mg/m^2^ showed lower leukemia-related and overall mortality than a lower dose of 80–110 mg/m^2^.
Aoki et al., 2016 (37)	RIC, 409	Evaluating the effectiveness of adding low-dose TBI to a RIC regimen in matched related HCT	The addition of low-dose TBI did not provide any benefit in terms of engraftment, relapse, NRM, or OS.
Yanada et al., 2020 (44)	MAC, 190 RIC, 284	Comparing long-term outcomes with RIC versus MAC allogeneic HCT for patients aged 50 years or older	Based on more than 10 years of follow-up data, RIC and MAC recipients had similar long-term outcomes regarding OS, relapse, and NRM.
Konuma et al., 2020 (47)	MAC, 652 RIC, 188	Comparing RIC and MAC allogeneic HCT for patients with poor cytogenetics in first CR	Patients receiving MAC had better OS and lower leukemia-related mortality than those receiving RIC.
Konuma et al., 2020 (52)	MAC, 732 RIC, 227	Evaluating transplant outcomes for patients with t(8;21)/inv(16) AML in relation to pretransplant MRD status	The conditioning intensity did not affect relapse or OS in patients with t(8;21) irrespective of the MRD status, whereas MAC provided better OS for those with inv(16) AML only if their pretransplant MRD was negative.
Konuma et al., 2022 (54)	MAC, 3580 RIC, 1907	Evaluating the survival trends for patients undergoing UCBT	RIC was associated with a higher risk of relapse-related mortality compared to MAC, whereas neither OS nor NRM differed between RIC and MAC.
Konuma et al., 2014 (56)	MAC, 438	Evaluating the effect of concurrent administration of G-CSF during conditioning on UCBT outcomes	G-CSF combined with CA/CY/TBI resulted in faster neutrophil engraftment, lower relapse, and better OS.
Shimomura et al., 2021 (64)	MAC, 846	Comparing FLU/BU4 and FLU/BU4/MEL for patients with various diseases including AML (71% of the total)	FLU/BU4/MEL showed a lower relapse rate and similar NRM, resulting in better OS compared to FLU/BU4.
Shimomura et al., 2021 (65)	MAC, 188	Comparing FLU/BU4/MEL and conventional MAC regimens for patients in non-CR.	FLU/BU4/MEL showed a lower relapse rate and similar NRM, resulting in better OS compared to conventional MAC regimens.
Mizuno et al., 2022 (66)	MAC, 477	Comparing FLU/BU4/MEL and CY/TBI with or without high-dose cytarabine for patients undergoing UCBT during CR.	FLU/BU4/MEL was associated with a lower risk of overall mortality compared to CY/TBI with or without high-dose cytarabine.

MAC, myeloablative conditioning; CY, cyclophosphamide; TBI, total body irradiation; BU, busulfan; iv, intravenous; NRM, non-relapse mortality; po, oral; UCBT, umbilical cord blood transplantation; OS, overall survival; BMT, bone marrow transplantation; PBSCT, peripheral blood stem cell transplantation; CR, complete remission; RIC, reduced-intensity conditioning; HCT, hematopoietic cell transplantation; FLU, fludarabine; MEL, melphalan; AML, acute myeloid leukemia; MRD, measurable residual disease; G-CSF, granulocyte-colony stimulating factor; CA, cytarabine.

## Author contributions

All authors wrote, edited, and approved the manuscript.

## Conflict of interest

The authors declare that the research was conducted in the absence of any commercial or financial relationships that could be construed as a potential conflict of interest.

## Publisher’s note

All claims expressed in this article are solely those of the authors and do not necessarily represent those of their affiliated organizations, or those of the publisher, the editors and the reviewers. Any product that may be evaluated in this article, or claim that may be made by its manufacturer, is not guaranteed or endorsed by the publisher.
